# A Prospective Study on the Influence of Scholastic Factors on the Prevalence and Initiation of Illicit Drug Misuse in Adolescence

**DOI:** 10.3390/ijerph15050874

**Published:** 2018-04-27

**Authors:** Zoran Zubak, Natasa Zenic, Ljerka Ostojic, Ivana Zubak, Haris Pojskic

**Affiliations:** 1 Faculty of Medicine, University of Mostar, 88000 Mostar, Bosnia and Herzegovina; zoran.zubak@gmail.com (Z.Z.); ljerka.ostojic@kifst.hr (L.O.); 2 Special Orthopedic Hospital Biograd n/m, 23210 Biograd, Croatia; 3 Faculty of Kinesiology, University of Split, 21000 Split, Croatia; 4 Academy of Medical Sciences, 71000 Sarajevo, Bosnia and Herzegovina; 5 Department for Ecology, Agronomy and Aquaculture, University of Zadar, 23000 Zadar, Croatia; izubak@unizd.hr; 6 Department for Health Sciences, Mid Sweden University, 83125 Östersund, Sweden; haris.pojskic@miun.se; 7 Swedish Winter Sports Research Centre, Mid Sweden University, 83125 Östersund, Sweden

**Keywords:** substance misuse, academic achievement, puberty, association

## Abstract

Background: This study aimed to prospectively investigate the scholastic factors related to illicit drug misuse (IDM) and the initiation of IDM among older adolescents from Bosnia and Herzegovina. Methods: This 2-year prospective study included 436 participants (202 females), who were an average of 16 years old at the beginning of the study (baseline). The participants were tested at baseline and follow-up (20 months later). The predictors included variables of scholastic-achievement (grade point average, school absences, unexcused absences and behavioral grade). The criteria were: (i) IDM at baseline; (ii) IDM at follow-up; and (iii) initiation of IDM over the study course. Results: Logistic regression indicated increased odds of IDM in adolescents who were more frequent absent from school (baseline: Odds Ratio (OR): 3.73, 95% Confidence Interval (CI): 2.12–6.57; follow-up: OR: 2.91, 95% CI: 1.90–4.65). The lower grade point average and more unexcused absences were evidenced for adolescents who consumed drugs on follow-up (OR: 1.67, 95% CI: 1.11–2.51; OR: 1.74, 95% CI: 1.30–2.32 for grade point average and unexcused absences, respectively). Initiation of IDM was predicted by frequent absences from school (OR: 2.2, 95% CI: 1.3–3.8), and lower behavioral grades (OR: 1.9, 95% CI: 1.2–3.3). Conclusions: The findings confirmed strong correlations between scholastic failure and IDM. Absences from school and lower behavioral grades at baseline were predictive of the initiation of IDM in older adolescents.

## 1. Introduction

Adolescence is considered a critical period for preventing substance misuse, which includes smoking, alcohol consumption, and misuse of illicit drugs. It is widely accepted that those individuals who do not start to consume substances before the age of 21 will likely never engage in such behavior later in life [1,2,3]. Although most teens do not escalate from trying drugs to developing addictive behaviors, even experimenting with substances is a problem. Namely, substance misuse can develop into a pattern of risky behavior including intoxicated driving, unsafe sex, injecting, and violent behavior [2,3,4] Therefore, even when adolescents do not develop symptoms of dependence (i.e., repeated substance misuse), even the rare usage of substances can pose a serious health-related risks and lead to socially dangerous behavior, with problems including family problems, failure in school (i.e., educational failure), altered interest in everyday healthy activities (i.e., proper eating, physical exercise), chemsex, mental health problems, impaired memory (i.e., problems with learning), risky sexual behavior with consequent increased risk of contracting infectious diseases (i.e., hepatitis C, HIV), etc. [5,6]. Not surprisingly, a great deal of public health efforts around the globe are oriented towards the prevention and reduction of substance misuse behaviors in adolescents [7,8,9,10,11].

Consumption of illicit drugs (marijuana, ecstasy, benzodiazepine, methadone, amphetamines, cocaine, heroin, etc.) is a highly specific type of substance misuse that occurs during adolescence. Briefly, in most countries, the illicit drug misuse (IDM) is considered illegal behavior. From the view point of preventive efforts, education, and public health, this is very important, since IDM is routinely associated with criminal behavior, which logically places those who misuse illicit drugs within the same context [12,13]. Therefore, special effort is needed to develop effective preventive campaigns against IDM in adolescents. One of the possible approaches is identification of the precipitating factors [14,15]. Briefly, the idea is to find those factors that are associated with consumption of illegal drugs; these either protective factors or factors that can contribute to increased risk for the IDM (i.e., risk factors). Identifying these factors will allow targeting of those adolescents who are at specific risk for IDM and consequently will assure the development of accurate and specifically tailored preventive programs. 

One group of factors that has been frequently studied in relation to the IDM in adolescents are familial (i.e., parental) factors [16,17,18,19,20]. Generally, studies have confirmed that specific parental styles and different parental factors are related to the IDM in children and adolescents. For example, an authoritative parenting style was found to be protective against the usage of ecstasy and cocaine [16], and the results consistently indicated that parental support, a higher level of parent-child communication and parental monitoring were protective against marijuana consumption [17,18,19,20,21]. Other factors that have been frequently studied in relation to the IDM are educational variables [15,22,23,24]. In brief, scholastic variables (i.e., educational achievements) are consistently related to the consumption of different illicit drugs, including inhalants (i.e., spray paints, glues, cleaning fluids), marijuana, and cocaine, with poorer scholastic achievements found in those adolescents who consume drugs [15,22,23,24]. 

Bosnia and Herzegovina (B&H) is a southeastern European country located in the Balkan Peninsula. Recent cross-sectional studies have identified a high prevalence of substance misuse in adolescents, placing the B&H among the European countries with the highest prevalence of adolescent substance misuse [25,26,27]. Because of these alarming figures, a consensus was reached about the necessity of developing an effective campaign against substance misuse in adolescents, including systematic evaluation of factors related to substance misuse. Consequently, previous cross-sectional studies have systematically investigated different groups of factors that are potentially related to cigarette smoking, alcohol consumption and the consumption of illicit drugs in this country. In terms of their findings related to IDM and related factors, parental factors (i.e., conflict with parents, parental monitoring, and parental concern) were strongly correlated with the usage of illicit drugs, with a higher likelihood for such behavior in those adolescents who reported certain problems in relationships with their parents and family [6,28,29]. Furthermore, poorer academic achievement was found in those adolescents who reported using illicit drugs [6,30]. However, the authors concluded that the relationship between scholastic achievement and substance misuse should not be simplified, since this relationship varies with respect to gender [28]. 

The main limitation of most studies that have investigated the relationships between certain precipitating factors and the misuse of illicit drugs, including those performed in B&H, is their cross-sectional design [28,29,30]. This approach allows interpreting the associations that exist between the variables, but causality remains unknown. Indeed, even the authors of the cited studies repeatedly concluded that prospective studies are necessary to objectively discuss the observed associations, which will consequently allow designing interventions targeting the enhancement of protective factors among young people at risk for substance misuse [29,30]. Therefore, the aim of this study was to prospectively investigate the relationship between scholastic achievement (predictors) and the use of illicit drugs (criterion) in 16–18-year-old adolescents from B&H. The goal was to investigate the relationship between the predictors and the criterion separately at two time-points (at the age of 16 and at the age of 18), and between the predictors and the initiation of IDM during the study. We hypothesized that the educational-variables observed at the beginning of the studied period (i.e., at the age of 16 years) would influence the initiation of IDM during the following two years (until the age of 18 years), with the higher likelihood for initiation among those adolescents with low scholastic achievement.

## 2. Materials and Methods

### 2.1. Pocedures and Participants

**Figure 1:**
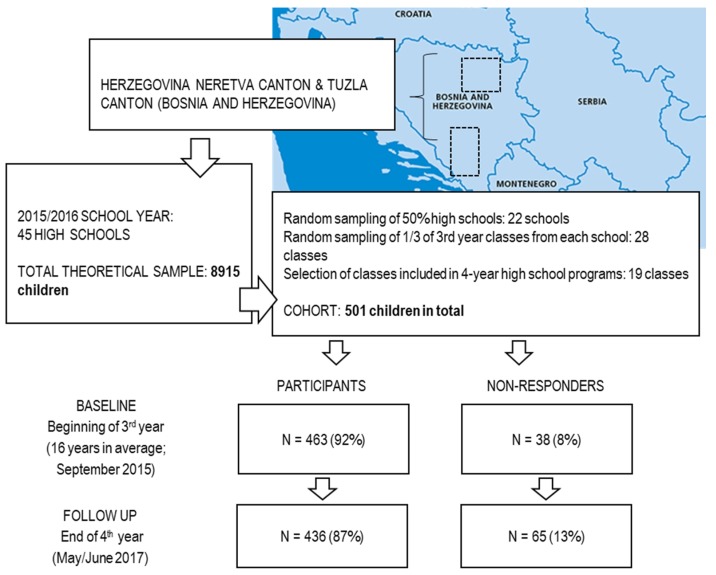
Location of the study, testing sequences, participant- and drop-out-rates.

In this study, we prospectively investigated adolescents during their last two years of high school (from 16–18-years of age). At baseline, the examinees were in their 3rd year of high school and were 16 years old on average. At the baseline, the sample of participants consisted of 501 adolescents, but in this study, we included 436 adolescents (202 females; 46%) who participated during both testing waves (see Figure 1 for the testing design and the number of respondents and drop-outs). Previous cross-sectional studies done on the territory of Bosnia and Herzegovina reported prevalence of illicit drugs consumption of approximately 10% [6,29]. On a basis of (i) such prevalence (e.g., 10%); (ii) population/theoretical sample of 8519 adolescents (Figure 1); and (iii) level of significance of *p* < 0.05; the sample of participants required for this study was 137 participants. For the purposes of selecting participants, we used a multistage simple random sampling method. 

During the first stage all the schools in the territories of Herzegovina-Neretva and Tuzla Canton in B&H were stratified by size into two groups. Next, one-third of all the 3rd year classes were selected via lottery from each group. Since we intended to study adolescents involved in 4-year high-school programs, in the next phase 19 classes included in 4-year programs were selected (Figure 1). The study personnel visited the selected classes during the first week of school year (in early September 2015) and explained the full procedure and study aims to the potential participants. Additionally, a written explanation was sent to the parents/guardians who were asked to sign a consent form for their children to participate in the study. The first test was conducted during the following two weeks (depending on the school). As a methodological remark, we must note that this study was one of the first to prospectively investigate scholastic variables as correlates of illicit drug consumption in southeastern Europe and was probably the first to examine this problem in the former territory of Yugoslavia. While previous cross-sectional studies have been performed in the territory following the same protocol of sampling and testing, we believe that the sample included in this investigation met the eligibility criteria necessary to objectively compare the results with those previously reported [6,14,29,30]. 

Participants were tested in the following two waves: baseline (beginning of the 3rd year of high school) and follow-up (end of the 4th year of high school, approximately 20 months after baseline testing). Testing was done during school hours in groups of at least 12 examinees. At the beginning of each testing wave, participants were informed that their participation was voluntary and that they could leave questions unanswered. Although the testing was anonymous, for identifying the test results across the two testing waves, participants used a self-selected confidential code (for ease of remembering, it was suggested that they use the last three digits of their e-mail password as their code). At the end of the testing, each participant placed the questionnaire in a closed box, which was opened next day, after testing at least three classes. The study was approved by the Ethical Board from the University of Split, Faculty of Kinesiology, Croatia (Approval No.: 2181-205-02-05-14-005; 11 September 2014). 

Before further statistical processing (see later text for details), the results were subjected to an analysis of attrition bias (Table S1). The intracluster correlation coefficient (for the baseline prevalence of illicit drug consumption, with the individual schools as clusters) was calculated. In brief, the attrition bias analysis showed no significant differences in the initial consumption of illicit drugs between those adolescents who remained in the study and those who dropped out (Chi square: 2.51, *p* = 0.11). There were significantly more males than females who dropped out (Chi square: 6.17, *p* = 0.01), but this was almost certainly related to fact that males were more often absent from school (including absence on testing days) [6]. Next, because of the known fact that similarity among participants within preexisting groups (i.e., clusters) reduces the variability of responses in a clustered sample, and consequently erodes the power to detect true differences between study arms we have calculated intracluster correlation coefficient (Rho). This was done for baseline illicit-use prevalence, with the individual schools as clusters. In short, the Rho was 0.06, indicating appropriate within-cluster (i.e., within-school) variance [31,32]. 

### 2.2. Variables

In this study, we evaluated a set of predictors, several covariates and criterion outcome measures. The predictors were factors related to scholastic achievement (educational factors). The outcome variable was the consumption of illicit drugs. Based on previous studies in which investigators repeatedly reported associations between gender, parental conflict, socioeconomic status (SES) and age, these variables were included as covariates [28,29,30,33]. The previously validated questionnaire (Questionnaire of Substance Use—QSU) was used for testing [6,34]. The complete questionnaire is provided in previous reports [6]. 

The educational variables included four questions assessing the following: (i) grade point average (GPA) during the last semester (participants responded on a five-point scale from excellent to negative); (ii) behavioral grade (four-point scale from excellent to under average); (iii) school absences (less than 10 school hours, 10–20 h, 21–40 h, or more than 40 h); and (iv) unexcused school absences (none, 1–5 h, 6–10 h, or more than 10 h). Although last two variables (i.e., (iii) and (iv)) are similar, different scales were used mainly because previous studies reported large differences in results between “total” and “unexcused” absences [6,34]. 

The scale for drug consumption included questions about the consumption of marijuana, hashish, heroin, cocaine, sedatives, and most party drugs (e.g., ecstasy, amphetamines). A seven-point range of consumption was offered for each question (ranging from “never” to “40 times and more”). Participants were later categorized in two groups. Specifically, those who have never used drugs were categorized as “non-users”, and others as “drug-users” [6]. 

Covariates (i.e., confounding factors) included participant age, gender, SES (under average–average–above average), and conflict with parents (four-point scale from “almost never” to “frequently”). 

### 2.3. Statistics

Descriptive statistics included counts and percentages. Differences between drug users and non-users in terms of the predictors and covariates were identified by the Mann-Whitney test (MW) or the chi-square test. To evaluate the relationship between the predictors and the outcome variable, a series of logistic regression models were generated, and odds ratios (ORs) and their corresponding 95% confidence intervals (CIs) were reported. The following three outcomes were evaluated: (i) consumption of illicit drugs at the study baseline; (ii) consumption of illicit drugs at follow-up; and (iii) the initiation of IDM over the course of the study. In addition to the crude logistic regression analysis (unadjusted regression models), the model adjusted for age, gender, SES and conflict with parents was also calculated. The logistic regression generated for the initiation of IDM included participants who were non-users at baseline. A *p*-value of 95% was applied. Statistica ver. 12.0 (Statsoft, Tulsa, OK, USA) was used for all the calculations.

## 3. Results

Overall, 4% of adolescents were identified as illicit drug users at the beginning of their 3rd year of high school, and 7% used illicit drugs at the end of high school 20 months later. The consumption of specific illicit drugs is presented in Figure S1. 

Table 1 presents the distribution of scholastic variables according to IDM status at baseline and follow-up. Non-users were less absent from school (MW: 4.29 (*p* < 0.01), 5.21 (0.01)) for baseline and follow-up, respectively), had better behavioral grades (2.02 (0.04), 4.72 [0.01], for baseline and follow-up, respectively), and had less unexcused absences at follow-up (4.51 (0.01)). At baseline, no significant differences between drug-users and non-users were evidenced for GPA, and number of unexcused absences. 

Drug-users and non-users did not differ in SES at baseline and follow-up. Also, there was no differences in drug-usage between genders at both testing waves. Those adolescents who did not use illicit drugs reported lower level of conflict with their parents-responsible adults (2.85 (0.01), 2.98 (0.01), for baseline and follow-up, respectively) (Table 2). 

Increased odds of illicit drug use were observed in adolescents who were more frequently absent from school (Baseline: OR: 3.73, 95% CI: 2.12–6.57; Follow-up: OR: 2.91, 95% CI: 1.90–4.65). Also, school absence at baseline was a significant predictor for initiation of IDM over the course of the study (OR: 2.21, 95% CI: 1.27–3.83). The lower GPA and more unexcused absences were evidenced for adolescents who consumed drugs on follow up measurement (OR: 1.67, 95% CI: 1.11–2.51; OR: 1.74, 95% CI: 1.30–2.32 for GPA and unexcused absences, respectively) (Table 3).

## 4. Discussion

This study aimed to prospectively investigate the potential relationships between scholastic factors with IDM in older adolescents. The analyses revealed some important findings that should be highlighted. First, scholastic factors were systematically associated with IDM, with poorer scholastic achievement in adolescents who reported illicit drugs use at baseline and follow-up. Second, behavioral grade, and absences from school at baseline were predictors of initiation of IDM in the following period. Therefore, the initial study hypothesis was confirmed. 

### 4.1. Scholastic Variables and the Consumption of Illicit Drugs 

The problem of the education-drug connection has been within the scope of the scientific and professional debates for more than five decades. While the first studies were mostly oriented towards identifying patterns of drug use in children and adolescents at different levels of schooling [33,34] and young people’s knowledge of drug use [35], later studies were more oriented towards identifying factors related to drug use [36], as well as those variables specifically explaining school success/failure (i.e., school performance, educational failure) [37]. Currently, interest in the relationship between educational success/failure and the consumption of illicit drugs is evident [38]. 

Our results showed specific relationships between scholastic variables (educational achievement) and the use of illicit drugs. In brief, in both the cross-sectional analyses (i.e., at baseline and follow-up), those adolescents who performed poorly in school were more likely to consume drugs; in terms of this matter, our results are consistent with previous studies on this problem [15,22,23,24,38]. However, the authors share the opinion that two findings on the association between scholastic variables and the consumption of drugs deserve special attention. First, the association between scholastic variables and drug consumption were much stronger for follow-up than for baseline testing; and second, the number of school absences was the strongest predictor of drug use both at baseline and follow-up-testing. 

Although studies have regularly confirmed a negative association between these variables [15,22,23,24,38], it seems that the strength of the relationship between scholastic variables and IDM in adolescents increased between the testing waves and was stronger at the end of the period of high school education. We were not able to find any study that examined this problem using the same experimental approach (i.e., calculating associations between educational variables and drug use at two-time points), and consequently, we were not able to compare our results with previous studies. However, based on the prevalence and dynamics of changes in studied variables, we can offer some specific explanations for our findings. 

Briefly, the prevalence of drug use over the course of the study evidently increased (from 4% to 7%). Consequently, for simple statistical reasons (i.e., a higher prevalence and, therefore, a more balanced number of participants in the “affected” and “non-affected” groups), it was more likely that logistic regression would reach statistical significance at follow-up than at baseline measurements. Additionally, the higher variability in educational achievements at the follow-up measurement could have influenced the statistical calculation similar to what was previously explained for the prevalence of drug consumption. 

Previous studies regularly used GPA as a general measure of students’ academic achievement, but our approach using some “non-standard” variables of scholastic achievement (e.g., behavioral grade, absences from school, unexcused absences from school) seems to be appropriate [39,40]. Indeed, the strongest predictor of drug consumption was “school absence”, and those students who reported more school absences (irrespective of whether those absences were formally recorded as “excused” or “unexcused”) were at risk of consuming illicit drugs at baseline and follow-up. These results additionally confirm our previous discussion on the possible background of the stronger correlation between the variables at the follow-up measurement. In brief, school absences must influence other educational measures (GPA mostly), but this takes time. Therefore, correlations between GPA and drug consumption were not identified until follow-up. 

Although studies have frequently examined the association between scholastic variables and the consumption of illicit drugs, the causality of this relationship is still relatively unknown [38]. Theoretically, it is possible that the consumption of drugs resulted in low academic achievement because of the negative effects of drugs on cognitive function, which consequently resulted in low learning capacity [41,42]. For example, smoking marijuana, which was the most frequently reported type of drug consumed in this sample (Figure S1), impairs short-term memory and attention. Consequently, one of the possible side-effects of marijuana usage is related to impaired educational attainment in adolescents [41,42,43]. 

Indeed, according to physiological influences of drugs on learning capacity, it seems reasonable to identify illicit drug consumption as the cause of low academic achievement in adolescents. However, it is also possible that causality should be interpreted based on the theory of social influence [44]. It is known that adolescents who are not well prepared for school duties often skip classes [45]. This puts them into “out-of-school” situations, where they are more likely to communicate with peers who consume substances, including those who use illicit drugs. Consequently, this increases the likelihood that they will start consuming drugs, as an effect of “low academic achievement” that results in out-of-school situations. Both explanations on the cause-effect relationship between the consumption of illicit drugs and low scholastic achievement are reasonable. Not surprisingly, the authors who examined these issues in cross-sectional studies repeatedly stated that for a more profound interpretation, prospective analyses are needed [6]. 

This is one of the first studies in southeastern Europe, and it is probably the first one in the former Yugoslav territory to prospectively examine the problem of the initiation of IDM in adolescents [1,14,34]. Therefore, our results on the significant influence of two educational variables (i.e., behavioral grade and school absence) on the initiation of drug use are novel to some extent. These findings have two important implications with respect to our study aims. First, results directly support our previous consideration of the applicability of “non-standard” educational variables for evaluating adolescents who are at a higher risk for consuming illicit drugs. Second, these findings clearly indicate that educational failure is the cause of the initiation of consumption of illicit drugs for adolescents over their last two years of high school education. While the first issue (i.e., the applicability of non-standard educational variables in evaluating at-risk adolescents) has already been discussed, in the following text, we will focus on the causality between scholastic variables and the initiation of drug use.

The variables of scholastic achievement that were found to be predictors of the initiation of drug use are specific. While the number of absences from school is a relatively objective measure, behavioral grade is a result of teachers’ personal evaluations of students’ behavior during a period of time (i.e., semester) [46]. From our standpoint, the fact that behavior grade was a significant predictor of the initiation of drug use is highly encouraging because it points to teachers’ high pedagogical competences. In brief, this type of evaluation of scholastic success/failure includes different non-formal indices of the students’ behavior, such as their attitude towards their peers and school staff, their approach to formal and non-formal school duties, and their involvement in extracurricular activities [46]. Behavioral grade is scored only by the class principal, who is the teacher who is responsible for one class per academic year, and in most cases, who follows the same class during all four years of high school. Throughout this period, class principals become familiarized with their classes, and obviously are capable of objectively recognizing different kinds of behavioral failures of their students. 

### 4.2. Limitations and Strengths

The main limitation of this study is that it was based on self-reported data. Therefore, participants may have tended to provide socially acceptable answers. However, we believe that the study design and our experience from previous studies in the protection of the anonymity of the participants reduced this possibility. Additionally, this study did not consider some important educational variables, such as college plans, which could be useful in the discussion and interpretation of the relationships between educational success/failure and drug consumption/initiation. Additionally, we should not ignore the possible effects of some confounders that were not observed in the investigation (e.g., parental educational status, parental employment, parental IDM). However, we believe that the variables included in this study as possible confounders (parental monitoring, SES) at least partially covered the aspects of the confounding indices that were not evaluated. 

This is one of the first studies conducted in southeastern Europe and probably the first one in the territory of former Yugoslavia to prospectively examine the association between educational and sport variables and drug consumption and initiation of drug use in adolescents. The prospective study design allowed not only the identification of this relationship but also the interpretation of the causality between the studied variables, which is an important strength of this investigation. 

## 5. Conclusions

The results showed strong associations between scholastic variables and illicit drug consumption in adolescents from B&H, and this study also showed the applicability of some “non-standard” measures of scholastic achievement as strong correlates of drug consumption. Namely, adolescents who were at risk for drug consumption had the lowest behavioral grades and were more absent from school. What is particularly important is that low behavioral grades at study baseline (i.e., at the end of the 2nd year of high school) were a significant predictor of the initiation of drug use during the study. Specifically, it seems that class principal teachers were able to objectively evaluate certain behavioral failures of their students, which in fact were correlated with their intention to use illicit drugs during the following period. As a result, teachers should be encouraged to use behavioral grade as a highly valuable measure and to inform parents and responsible adults about their evaluations regarding this indicator of educational success. 

All the studied predictors of IDM and initiation of IDM are easily obtainable through the regular scholastic system. Therefore, in developing effective and accurate preventive efforts against illicit drug use, here evidenced factors associated to higher risk should be considered. School and public health authorities should be informed about our findings to include these information in specific school-based educational programs against IDM.

## Figures and Tables

**Table 1:** Baseline and follow-up results for scholastic variables (F—frequency, %—percentage) with differences on a basis of consumption of illicit drugs (MW—Mann Whitney Z values, *p*—level of significance).

Predictors	Baseline			Follow-Up		
	Nonusers	Users	MW	Nonusers	Users	MW
	F (%)	F (%)	Z (*p*)	F (%)	F (%)	Z (*p*)
**Grade point average**						
Excellent	160 (38.37)	5 (26.32)	−0.60 (0.54)	156 (38.33)	8 (27.59)	−2.03 (0.04)
Very good	156 (37.41)	10 (52.63)		162 (39.8)	9 (31.03)	
Average	83 (19.9)	3 (15.79)		81 (19.9)	10 (34.48)	
Under average	6 (1.44)	0 (0)		5 (1.23)	0 (0)	
Poor	12 (2.88)	1 (5.26)		3 (0.74)	2 (6.9)	
Missing	0 (0)	0 (0)		0 (0)	0 (0)	
**Absences from school**						
Less than 10 h	188 (45.08)	2 (10.53)	−4.29 (0.01)	147 (36.12)	1 (3.45)	−5.21 (0.01)
10–20 h	163 (39.09)	6 (31.58)		165 (40.54)	8 (27.59)	
21–40 h	54 (12.95)	7 (36.84)		77 (18.92)	16 (55.17)	
More than 40 h	12 (2.88)	4 (21.05)		18 (4.42)	4 (13.79)	
Missing	0 (0)	0 (0)		0 (0)	0 (0)	
**Unexcused absences**						
None	300 (71.94)	12 (63.16)	−1.00 (0.31)	276 (67.81)	9 (31.03)	−4.51 (0.01)
1–5 h	75 (17.99)	3 (15.79)		78 (19.16)	8 (27.59)	
6–10 h	19 (4.56)	2 (10.53)		30 (7.37)	5 (17.24)	
More than 10 h	23 (5.52)	2 (10.52)		23 (5.56)	6 (20.68)	
Missing	0 (0)	0 (0)		0 (0)	1 (3.45)	
**Behavioral grade**						
Excellent	359 (86.09)	13 (68.42)	−2.02 (0.04)	344 (84.52)	15 (51.72)	−4.72 (0.01)
Very good	38 (9.11)	5 (26.32)		32 (7.86)	4 (13.79)	
Average	11 (2.64)	1 (5.26)		22 (5.41)	7 (24.14)	
Under average	9 (2.16)	0 (0)		6 (1.47)	1 (3.45)	
Poor	0 (0)	0 (0)		3 (0.74)	2 (6.9)	
Missing	0 (0)	0 (0)		0 (0)	0 (0)	

**Table 2:** Baseline and follow-up results (F—frequency, %—percentage) for socioeconomic status, gender, and parental conflict (covariates) with differences on a basis of consumption of illicit drugs (MW—Mann Whitney Z value, Chi square test—Chi^2^, *p*—level of significance).

Covariates	Baseline			Follow-Up		
	Nonusers	Users	MW	Nonusers	Users	MW
	F (%)	F (%)	Z (*p*)	F (%)	F (%)	Z (*p*)
**Socioeconomic status**						
Under average	6 (1.44)	1 (5.26)	0.61 (0.54)	12 (2.95)	4 (13.79)	0.79 (0.47)
Average	389 (93.29)	17 (89.47)		382 (93.86)	22 (75.86)	
Above average	22 (5.28)	1 (5.26)		13 (3.19)	3 (10.34)	
Missing	0 (0)	0 (0)		0 (0)	0 (0)	
**Conflict with parents/responsible adults**						
Almost never	170 (40.77)	3 (15.79)	−2.85 (0.01)	195 (47.91)	6(20.69)	−2.98 (0.01)
Rarely	161 (38.61)	8 (42.11)		152 (37.35)	15(51.72)	
Occasionally	79 (18.94)	4 (21.05)		54 (13.27)	6(20.69)	
Frequently	7 (1.68)	4 (21.05)		6 (1.47)	2(6.9)	
Missing	0 (0)	0 (0)		0 (0)	0(0)	
			**Chi^2^** **(*p*)**			**Chi^2^ (*p*)**
Gender						
Male	222 (53.24)	8 (42.11)	0.99 (0.31)	213 (52.33)	12 (41.38)	1.47 (0.23)
Female	191 (45.8)	11 (57.89)		190 (46.68)	17 (58.62)	
Missing	4 (0.96)	0 (0)		4 (0.98)	0 (0)	

**Table 3:** Crude and adjusted logistic regression models of the relationship between independent variables and illicit drug misuse at baseline and follow-up, and initiation of illicit drug misuse during the study course, OR with 95% confidence interval (CI).

Predictors	Baseline OR (95% CI)	
	Crude	Adjusted
Grade point average ^cont^	1.14 (0.72–1.80)	1.21 (0.76–1.92)
Absences from school ^cont^	3.18 (1.91–5.30)	3.73 (2.12–6.57)
Unexcused absences ^cont^	1.26 (0.85–1.87)	1.40 (0.91–2.14)
Behavioral grade ^cont^	1.61 (0.86–3.02)	1.40 (0.77–2.54)
	**Follow-Up OR (95% CI)**	
	**Crude**	**Adjusted**
Grade point average ^cont^	1.66 (1.11–2.47)	1.67 (1.11–2.51)
Absences from school ^cont^	3.00 (1.92–4.69)	2.91 (1.90–4.65)
Unexcused absences ^cont^	1.72 (1.30–2.27)	1.74 (1.30–2.32)
Behavioral grade ^cont^	2.08 (1.50–2.88)	2.14 (1.53–3.02)
	**Initiation ^¥^ OR (95% CI)**	
	**Crude**	**Adjusted**
Grade point average ^cont^	1.53 (0.98–2.39)	1.50 (0.94–2.39)
Absences from school ^cont^	2.38 (1.38–4.13)	2.21 (1.27–3.83)
Unexcused absences ^cont^	1.46 (0.99–2.14)	1.34 (0.90–1.98)
Behavioral grade ^cont^	2.13 (1.29–3.52)	1.93 (1.15–3.27)

Crude—non-adjusted logistic regression; Adjusted—logistic regression adjusted for age, gender, socioeconomic status and conflict with parents, ^cont^ variables were observed as continuous for logistic regression calculations, ^¥^ participants who were users of illicit drugs at study baseline were not included in this analysis.

## Supplementary Materials

The following are available online at http://www.mdpi.com/1660-4601/15/5/874, Figure S1: Prevalence of illicit drug misuse at study baseline and follow-up, Table S1: Attrition bias analysis between responders and non-responders on a basis of illicit drug use at baseline and gender.

## Abbreviations

The following abbreviations are used in this manuscript:

GPA	grade point average
SES	socioeconomic status
B&H	Bosnia and Herzegovina
