# Supplementary material for: A Prospective Study on the Influence of Scholastic Factors on the Prevalence and Initiation of Illicit Drug Misuse in Adolescence
Source: Int J Environ Res Public Health. 2018 Apr 27;15(5):874. doi: 10.3390/ijerph15050874 (PMC5981913; doi:10.3390/ijerph15050874)
Supplement: Supplementary file 1 [file ijerph-15-00874-s001.pdf]

Supplementary Materials

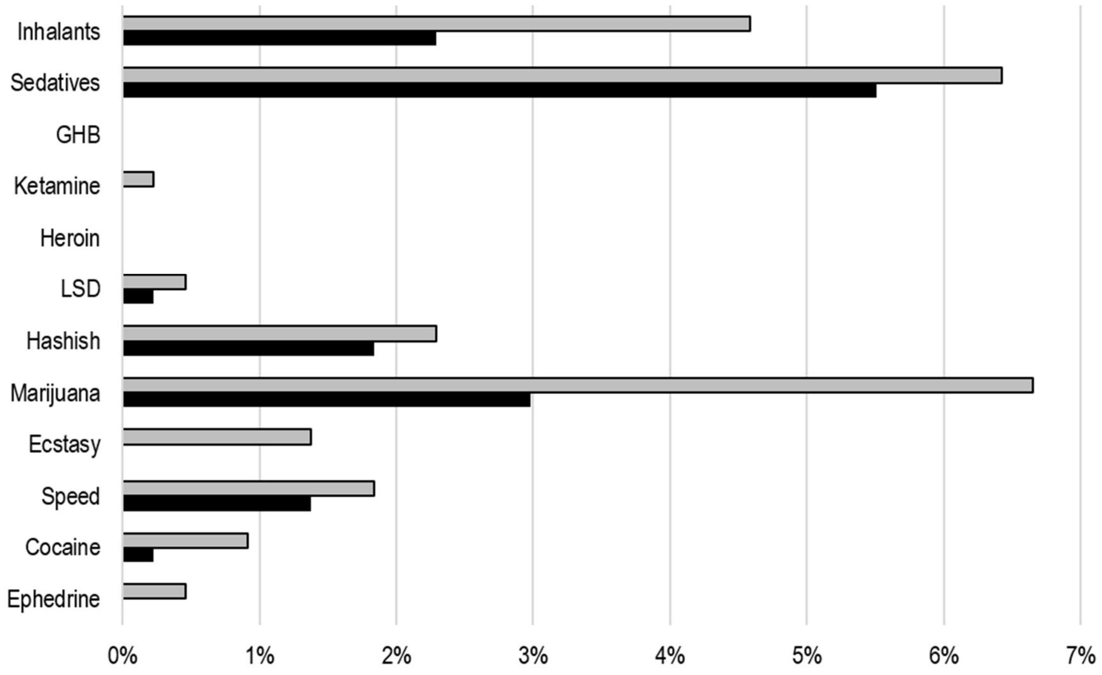

	Ephedrine	Cocaine	Speed	Ecstasy	Marijuana	Hashish	LSD	Heroin	Ketamine	GHB	Sedatives	Inhalants
Follow-up	0.5%	0.9%	1.8%	1.4%	6.7%	2.3%	0.5%	0.0%	0.2%	0.0%	6.4%	4.6%
Baseline	0.0%	0.2%	1.4%	0.0%	3.0%	1.8%	0.2%	0.0%	0.0%	0.0%	5.5%	2.3%

Prevalence (%)

Figure S1. Prevalence of illicit drug misuse at study baseline and follow-up.

Table S1. Attrition bias analysis between responders and non-responders on a basis of illicit drug use at baseline and gender.

	Responders	Non-Responders	Chi Square (p)
Illicit drug users			
Males	8	2	0.63
Females	11	1	(0.42)
Illicit drug nonusers			
Males	222	19	5.92
Females	191	5	(0.02)
Subtotal illicit drug users	19	3	2.51
Subtotal illicit drug nonusers	413	24	(0.11)
Subtotal males	230	21	6.17
Subtotal females	202	6	(0.01)
Total	432	27	

Note that group of non-responders for this analysis does not include participants who were not present at the baseline testing (38 adolescents) and 4 participants who did not specify gender.
